# Impact of Synchronous Online Learning on the Theoretical Training of New Pediatric Nurses: A Prospective Randomized Trial

**DOI:** 10.7759/cureus.76783

**Published:** 2025-01-02

**Authors:** Shaodan Qi, Xiaojing Hu, Ying Gu, Mengxin Zhu

**Affiliations:** 1 Nursing, Children's Hospital of Fudan University, Shanghai, CHN

**Keywords:** clinical nursing, novice pediatric nurses, pre-service training, randomized controlled trial, synchronous online learning, theoretical training

## Abstract

Aims

This study aimed to examine the impact of synchronous online learning on the effectiveness of theoretical training and self-efficacy among newly graduated pediatric nurses.

Design

It is a prospective randomized controlled trial.

Methods

The sample comprised 45 newly graduated pediatric nurses randomly assigned to an experimental cohort group or a cohort group. The control group underwent traditional face-to-face teaching, whereas the experimental group engaged in synchronous online learning. A comparative analysis encompassed the test scores from post-session 10-minute assessments (T2), evaluations conducted after one week (T3), and those after one month (T4), alongside their academic self-efficacy scores.

Results

Forty-five newly graduated pediatric nurses participated with 23 allocated to the control cohort and 22 allocated to the experimental cohort. The baseline characteristics revealed no statistically significant disparities between the two groups. The post-session 10-minute assessment (T2) revealed a slight, yet not statistically significant, advantage for the experimental group over the control group. However, in contrast, the experimental cohort exhibited significantly superior performance compared to the control group during theoretical evaluations conducted one week (T3) and one month (T4) after training (p<0.001). In terms of academic self-efficacy, the experimental group demonstrated significantly higher scores than their counterparts in the control group.

Conclusions

Synchronous online learning that is meticulously structured and closely monitored has the potential to enhance the efficacy of theoretical training and boost academic self-efficacy among new graduate nurses.

## Introduction

Nursing students are expected to acquire the necessary skills and knowledge in nursing science and theory during their education, in order to practice as registered nurses [1]. However, due to insufficient clinical instruction, exposure, and workplace readiness, new graduate nurses exhibited lower confidence levels when entering clinical work [2]. The transition from university-based nurse education to hospital-based programs led to the introduction of graduate programs for newly qualified registered nurses aimed at facilitating their assimilation into the clinical environment [3]. This training is crucial for career progression and developing proficiency as clinical nurses. As an integral part of post-graduation professional education, standardized training for novice nurses plays a pivotal role in academic learning and practical experience while serving as the foundation of clinical nursing pedagogy. With advancements in technology, there is a growing trend towards web-based training. Online learning has the potential to overcome limitations related to numbers, geography, and space [4]. Particularly during situations such as the COVID-19 pandemic where reducing person-to-person contact is essential, online learning becomes indispensable. However, debates regarding its efficacy persist. Therefore, this study aims to examine the outcomes of synchronous online learning for novice pediatric nurses through a randomized controlled study design.

The World Health Organization (WHO), in collaboration with the International Council of Nurses (ICN), reported that there were nearly 28 million nurses globally in 2020, yet there remains a shortage of approximately 5.9 million nurses [5]. A comparative analysis revealed that turnover rates for new graduate nurses within their first year of employment were approximately 26.8% in the United States, 19.9% in Canada, 44.3% in New Zealand, and 15.1% in Australia [6]. Due to the increasing nursing shortage, there is a demand for new graduate nurses with less than one year of nursing experience to fill these gaps; however, many new graduate nurses worldwide leave their initial position within the first year. The transition from student to qualified nurse has long been acknowledged as a stressful experience. While 90% of deans believed that their nursing students were fully prepared to deliver safe and effective patient care, only 10% of nurse leaders shared this belief [7]. The complex and fast-paced acute care environment encountered by new graduate nurses presents numerous challenges. Educational interventions for new graduate nurses and revisions to nursing curricula have been suggested as strategies to address clinical challenges and potentially enhance job satisfaction. The teaching of new graduate nurses has become an important responsibility for hospital nursing managers.

In response to the COVID-19 pandemic, educational institutions worldwide have witnessed an increase in remote, distance, and blended learning approaches. These digital teaching and learning methods have emerged as alternatives to traditional face-to-face instruction. Synchronous online learning utilizes videoconferencing and messaging systems to facilitate real-time interaction between students, requiring simultaneous presence for effective teaching. While synchronous online learning may appear to impose similar temporal constraints as face-to-face instruction, its unique advantage lies in the ability to provide immediate educational support and feedback compared to asynchronous learning [8]. The logistical benefit of synchronous online learning is the opportunity for participants to engage in the learning process from any geographical location. An instructional advantage is the potential for learners to access rich multimedia resources. Furthermore, there are financial benefits associated with synchronous online learning such as eliminating travel costs and reducing time away from clinical training sites while still enabling real-time interaction between students and instructors [9].

## Materials and methods

Study design

This is a single-center, prospective randomized controlled trial conducted at the National Children's Medical Center in China. The trial compared the effectiveness of traditional face-to-face teaching and synchronous online learning in training novice nurses. This study was approved by the Ethics Committee of the Children's Hospital of Fudan University (approval number: (2018) 211) and was also registered at ClinicalTrials.gov (NCT06650020). Participants provided informed consent before participating in the study.

Study criteria

The study sample consisted of novice nurses recruited in 2022 from our institution, a tertiary-level children's hospital designated as Class A. Inclusion criteria included the following: (1) continued engagement in full-time nursing studies and (2) successful completion of pre-employment training evaluation at the hospital. In contrast, exclusion criteria were defined as follows: (1) inability to complete all specified training modules and (2) unwillingness to participate in the study.

Study procedure

Establishing a Specialized Teaching Team

The deputy director of the nursing teaching and research department assumed the role of team leader, overseeing a specialized teaching group consisting of 12 esteemed clinical educators. The team leader was responsible for orchestrating, managing, and ensuring quality control of training initiatives, while the teaching officer executed training with precision and integrated all aspects of the process. Together, the team of educators took on responsibilities including curriculum design, instructional delivery, and formulation of examination propositions.

Forming a Team of Educators

The selection process for the teaching team involved a meticulous evaluation of our college's full-time teaching staff, encompassing diverse specializations. Eligibility criteria included a bachelor's degree or higher qualification, a minimum of 10 years' professional experience, holding a nursing teaching position of grade N3 or above, demonstrating strong professional ethics and conduct, a heightened sense of duty, passion for the teaching profession, proficiency in teamwork and service orientation, effective communication and organizational skills, and unwavering commitment to ethical standards and academic rigor. Additionally, candidates were expected to have thorough knowledge of relevant policies, systems, and training standards related to nursing education as well as profound theoretical understanding informed by substantial clinical exposure. This comprehensive expertise was complemented by proficiency in standardized clinical practices supported by demonstrable teaching ability and pedagogical finesse.

Refining the Development of Training Content

The foundational body of training content was organized in accordance with the Outline, covering nine distinct domains: legislative frameworks, normative benchmarks, regulatory paradigms, safety management, nursing documentation, health education, psychological nursing, communication skills, and professional ethics. The realm of professional theory included four main dimensions: domain-specific knowledge, specialized techniques, health counseling, and allied awareness. Furthermore, by integrating the findings from the 2022 survey on training needs for novice nurses and feedback from trainees in 2021, additional themes such as pediatric anatomy, humanistic care, vocational ethics, and professional development skills were incorporated into the theoretical framework. This expansion has enhanced the breadth and depth of the training program. A curriculum consisting of 12 modules was implemented, with each module spanning 1.5 hours.

Designing Online Courses

The theoretical framework of the Online Collaborative Inquiry Community (COI), developed by Randy Garrison, a prominent Canadian scholar in education, represents a robust pedagogical theory in the field of online and blended learning. This framework encompasses the enhancement of cognitive presence, social presence, and teaching presence. At its essence, it emphasizes the interconnected relationship among these three elements: cognitive existence, social interaction, and instructional engagement. This dynamic convergence serves to instill trainees with the essence of inquiry-based learning throughout their online educational journey. It not only enhances the learning experience but also promotes deep and meaningful comprehension, thereby stimulating the development of higher-order cognitive faculties such as critical and innovative thinking [10]. The ongoing investigation, based on the COI model and focusing on the cognitive dimension, has developed an online pedagogical approach consisting of four distinct exploratory phases: initiation of triggering events, collective exploration, integration of insights, and formulation of solutions. For example, in the module "Peripheral Venous Indwelling Needle Management," the sequence unfolded as follows: First, in Triggering Event, the instructor introduced the central inquiry as "strategies for selecting blood vessels for pediatric peripheral vein indwelling," prompting cognitive engagement. Second, in Exploration, trainees engaged in group deliberations regarding vessel selection criteria, its pros and cons, while also sharing insights with peers. Third, in Integration, facilitators intervened with timely feedback, amalgamating trainees' reflections and peer inputs to converge on optimal solutions. Lastly, in Solution, instructors utilized anatomical atlases and three-dimensional visual aids to elucidate pediatric vascular structures, accompanied by clinical case studies to expound on vessel selection during pediatric peripheral vein retention, thereby inspiring practical application among trainees.

Enhancing the Pedagogical Implementation of Two Distinct Cohorts

Group allocation: Group allocation was conducted using a computer-generated random number table, with trainees divided into a control group (receiving face-to-face instruction) and an experimental group (engaging in synchronous online learning) at a 1:1 ratio. The allocation process was administered by independent researchers, who concealed the outcomes within sealed envelopes. The names of the trainees were meticulously recorded on the envelopes to ensure the integrity of allocation, and only after a comprehensive evaluation of baseline data were the envelopes unsealed to determine group assignments. The control group consisted of 23 individuals receiving conventional face-to-face instruction, while the experimental group comprised 22 individuals participating in synchronous online learning.

Training methods for the control group: The control group adhered to the traditional offline model of centralized training, which involved a training facility spanning 100 square meters and accommodating seating for 150 participants. The front section of the training room was equipped with various amenities, such as a projection screen, podium, computer, and microphone, all available for use by the instructors. Throughout the training period, the lecturer provided in-person instruction on-site, allowing trainees to engage in real-time interaction by raising their queries.

Training methodologies employed in the experimental cohort: The experimental group implemented an online training modality. Each participant in this cohort selected a suitable and peaceful learning environment, characterized by ample lighting and strong network connectivity. Equipped with laptops, participants logged into the Tencent conference software 10 minutes prior to the session, entered their authentic names, completed the sign-in process five minutes before the course started, and kept their cameras activated throughout the session. During the instructional phase, both the teacher and control group were physically present. While the teacher instructed the control group, screen sharing through Tencent conference software allowed trainees in the experimental group to have clear visual access to instructional materials while audibly receiving instruction through microphones. Importantly, throughout the teaching session, trainees were encouraged to activate their microphones at any time for unrestricted query resolution.

Sample size calculation

The determination of the sample size was based on a preliminary investigation and a review of similar prior research experiences. Anticipating a 13% difference between the experimental and control groups, with α set at 0.05 and β at 0.10, and factoring in a dropout rate of 10%, each group was projected to consist of 18 participants.

Tools and assessments

Theoretical Evaluation

The examination paper was an internally developed document, with the instructor independently formulating the topics relevant to the course. The content was carefully aligned with the course's instructional material and underwent rigorous review by the teaching team. Each unit of course content was accompanied by five corresponding questions, including two single-choice, two multiple-choice, and one judgment-based query. Overall, the examination comprised 60 questions, each worth 2 points, resulting in a total of 120 points. The assessment occurred across multiple stages: the pre-class test (T1), the 10-minute post-class test (T2), the test after one week (T3), and the test after one month (T4). Organized by the teaching officer of the nursing teaching and research department, exam papers were randomly generated through a computer program and consistently distributed via an online nursing training platform. While identical papers were used for exams, question arrangement was randomized for each instance. Following class sessions, T2 was administered as an online examination for the experimental group and as an offline test for the control group. T1, T3, and T4 assessments were all conducted offline at a central location under the supervision of a teaching officer. In the case of the online T2 examination, trainees kept their cameras and microphones activated throughout the process with another teaching officer monitoring the session. At the start of the exam, participants logged into the app and accessed the test paper with a total allotted time of 40 minutes. Once initiated, the test automatically triggered submission upon any program switch.

Assessment of Academic Self-Efficacy

The evaluation questionnaire, developed by Liang from Central China Normal University [11], was inspired by the relevant dimensions in Pintrich and De Groot's academic self-efficacy questionnaire [12]. It was divided into two domains, learning aptitude self-efficacy and learning behavioral self-efficacy, comprising a total of 22 test items. Learning aptitude self-efficacy refers to an individual's assessment and confidence in their ability to effectively navigate their studies, achieve commendable outcomes, and avoid academic shortcomings. The scoring methodology utilizes a five-point scale, where "completely disagree" corresponds to 1 point, "relatively disagree" corresponds to 2 points, "uncertain" corresponds to 3 points, "relatively agree" corresponds to 4 points, and "completely agree" corresponds to 5 points. Within this structure, questions 14, 16, 17, and 20 are subject to reversed scoring. A higher cumulative score indicates increased academic self-efficacy. The combined total of the two dimensions represents the overall academic self-efficacy score. Similarly, a higher score indicates a heightened level of self-efficacy. The questionnaire is intended to be administered by the teaching officer one month after the class session specifically at T4; it will be distributed and collected in person during this period.

Statistical analysis

The data analysis was performed using IBM SPSS Statistics for Windows, Version 20.0 (Released 2011; IBM Corp., Armonk, New York, United States). Measurement data with a normal distribution were presented as mean±standard deviation (SD). The two-sample independent t-test was utilized for comparing such data. Non-normally distributed data were expressed as median (interquartile range (IQR)) and compared using the Mann-Whitney U test. Categorical data were analyzed using the χ2 test or Fisher's exact test as appropriate. A significance level of p<0.05 was used to indicate statistical significance.

## Results

Demographics

A comparison of general information between the two groups of new nurses is presented in Table 1. A total of 45 out of 50 newly recruited nurses met the inclusion criteria over a period of 22 years, with 22 assigned to the control group and 23 to the experimental group. One nurse withdrew from the study due to illness after the theoretical examination in January. There were no statistically significant differences in baseline characteristics between the two cohorts (p>0.05). Please refer to Table 1 for detailed information.

**Table 1:** Comparative analysis of general data between the two groups

Item		Control group (n=22)	Experimental group (n=23)	Statistical value	P-value
Gender, n (%)	Male	1 (4.35%)	2 (9.09%)	0.407	0.524
	Female	22 (95.65%)	20 (90.91%)		
Age, years		22.30±1.06	22.95±1.25	-1.677	0.094
Education background, n (%)	Master	1 (4.35%)	0 (0%)	1.360	0.506
	Undergraduate	14 (60.87%)	14 (63.64%)		
	College	8 (3.48%)	8 (18.18%)		
Political outlook, n (%)	Party member	4 (17.39%)	1 (4.55%)	2.135	0.344
	League member	17 (73.91%)	18 (8.18%)		
	General public	2 (8.70%)	3 (13.64%)		
Only child, n (%)	Yes	11 (47.83%)	16 (72.73%)	2.905	0.088
	No	12 (52.17%)	6 (27.27%)		
Served as a trainee cadre during college, n (%)	Yes	13 (56.52%)	13 (59.09%)	0.034	0.862
	No	10 (43.48%)	9 (40.91%)		
Pre-class test (T1), points		73.30±9.97	72.19±10.47	0.361	0.720

Theoretical evaluation scores

In the control group, one nurse was unable to participate in the post-class month test (T4) assessment due to sick leave, while the remaining assessments were successfully completed. The comparative analysis of theoretical examination scores between the two groups revealed significantly higher scores for the experimental group in both the one-week test (T3) and the one-month test (T4) after the class (p<0.001). For more detailed information, please refer to Table 2.

**Table 2:** Comparative analysis of theoretical scores between the two groups (scores: M (Q25, Q75)) ^1^: T value; ^2^: Z value; T1: pre-class test; T2: 10-minute test after class; T3: one-week test after class; T4: one-month test after class

Group	Control group		Experimental group		Statistical value	P-value
	Number	Assessment scores	Number	Assessment scores		
T1	22	73.30±9.97	23	72.19±10.47	0.361^1^	0.72
T2	22	111.91±8.43	23	115.45±4.63	-1.736^1^	0.088
T3	22	114 (104, 116)	23	118 (114, 120)	-25.969^2^	<0.001
T4	21	114 (108, 116)	23	118 (112, 118)	-23.994^2^	<0.001

Scores of academic self-efficacy

The comparison of academic self-efficacy scores between the two new nurse groups revealed that the experimental group demonstrated significantly higher scores in learning behavior self-efficacy and academic self-efficacy compared to the control group (p<0.05). Please refer to Table 3 for detailed results.

**Table 3:** Comparative analysis of total scores and scores across various dimensions of academic self-efficacy (scores, x̄±s) *: p<0.05

Item	Control group (n=21)	Experimental group (n=23)	T value	P-value
Learning ability and self-efficacy	Version 4.04±0.63	Version 4.26±0.46	1.323	0.194
Self-efficacy of learning behavior	Version 3.52±0.35	Version 3.95±0.66	2.701	0.011^*^
Total score of academic self-efficacy	Version 3.78±0.44	Version 4.10±0.48	2.313	0.026^*^

## Discussion

Synchronous online learning is a form of online education characterized by immediate communication and interaction among participants within a text-based or video-based environment. This includes learning activities taking place through mediums such as chat rooms or video conferences [13]. In contrast to traditional face-to-face instructional methods, synchronous online learning allows both learners and educators to access the learning process remotely from different locations around the world, enabling simultaneous participation. Advances in online learning technologies, including audio, video, and text mediums, enable prompt feedback and real-time engagement among learners, instructors, and peers [14]. In recent years, synchronous online learning has been increasingly used across various educational domains [14]. Research findings [10,15] highlight increased learner satisfaction with online learning. Its main advantages include saving travel time, providing flexible formats for individualized pacing, enhancing comfort, and reducing costs [16]. At the same time, the online environment improves accessibility especially for those who are reluctant to participate in on-site lectures. Individuals who may feel more reserved in traditional classrooms tend to be more comfortable asking questions and expressing their views within the online sphere. Therefore, synchronous online learning emerges as an educational paradigm that meets contemporary requirements and is better aligned with learners' expectations: a trend that is relevant today.

Online learning has emerged as a crucial instructional approach in medical education, with practical applications. However, it is more than just a digital adaptation of traditional face-to-face teaching; rather, it requires an adapted version of current teaching practices [17]. Therefore, it is essential to integrate well-structured design and pedagogical strategies into online coursework. This study implemented a synchronous online educational model, which involved sign-ins, camera activation, and interactive questioning during instructional sessions. The course design was carefully aligned with the COI model to provide structure and facilitate effective teaching activities. In terms of theoretical assessment, the experimental group's performance on the pre-class test (T1) showed slightly lower results compared to the control group. However, after the post-class 10-minute test (T2), the experimental group exhibited a slight advantage over the control group, although this difference was not statistically significant (p>0.05). Essentially, there was no significant lag in theoretical assessment outcomes for the online training cohort compared to the control group. Interestingly, during theoretical assessments conducted one week (T3) and one month (T4) after class, the experimental group outperformed the control group (p<0.05). This difference can be attributed to trainees undergoing traditional centralized training often having to contend with morning commutes to hospitals or classrooms and potentially arriving fatigued and susceptible to peer influences. Based on these findings, it is evident that synchronous online learning supported by structured design enhances theoretical training efficacy for new pediatric nurses.

Self-efficacy refers to an individual's belief in their ability to perform specific work behaviors using their skills. It encompasses self-confidence towards particular tasks, forming a subjective sense of personal capability, an influential factor affecting learning behaviors (e.g., problem-solving, autonomous learning), cognitive processes, and emotional states [18,12]. Importantly, self-efficacy has a positive psychological impact on problem-solving and enhances the effectiveness of issue resolution. In this study, the experimental group exhibited higher scores in both learning behavior self-efficacy and academic self-efficacy compared to the control group (p<0.05). Unlike traditional face-to-face instruction which is closely monitored by teachers, online learning provides trainees with a more relaxed and independent environment that allows them greater freedom in determining their learning approaches. Research suggests that conventional rigid teaching methods during in-person instruction may struggle to stimulate trainee interest, leading to reduced initiative and overall teaching efficacy [18]. Furthermore, 92.9% of trainees expressed more enthusiasm for online learning than traditional methods [19]. Online teaching can enhance learning motivation and self-efficacy [20,21]. Clearly, synchronous online learning creates an environment conducive to enhancing self-efficacy and fostering autonomous learning capabilities.

Particular emphasis was placed on the meticulous oversight of online teaching throughout the research process. It is noteworthy that inadequate management of online teaching could lead to varied outcomes. Therefore, effective online teaching requires a rigorous focus on scientific curriculum design and strict pedagogical administration, including the cultivation of students' enthusiasm for online learning, in order to foster improved results.

Limitations

This study represents the first randomized controlled trial to assess the theoretical training of pediatric novice nurses through synchronous online learning. The intervention and control groups were randomly selected from the same clinical trial population. The primary outcome indicators of the trial were collected at multiple time points and were objective in nature. However, blinding of participants and instructors was not feasible, and the generalizability of the results may be limited by the single-center design.

## Conclusions

Synchronous online learning presents numerous advantages and potential benefits for both educators and learners. It is entirely feasible to deliver education of comparable quality through web-based resources, potentially leading to equivalent or superior outcomes. However, the lack of flexibility in timing and the increased demands on curriculum design and technical infrastructure pose a challenge to enhancing learners' engagement in synchronous online learning. Several issues require resolution and further exploration.
